# First records and new information on the associations of echinoderms with other phyla in the rocky reefs of northern Chocó, Colombian Pacific

**DOI:** 10.3897/zookeys.921.32802

**Published:** 2020-03-24

**Authors:** María Juliana Vanegas González, Giomar Helena Borrero-Pérez

**Affiliations:** 1 Instituto de Investigaciones Marinas y Costeras José Benito vives de “Andreis” – INVEMAR. Calle 25 No. 2–55, Playa Salguero, Rodadero, Santa Marta, Colombia Instituto de Investigaciones Marinas y Costeras José Benito vives de “Andreis” Santa Marta Colombia

**Keywords:** Commensalism, epibionts, parasitism, Asteroidea, Echinoidea, Holothuroidea, Ophiuroidea, Riscales

## Abstract

Rocky reefs of the northern Colombian Pacific (Chocó) are diverse ecosystems that are poorly studied. Echinoderms are one of the principal groups in these ecosystems with associations to different species, including benthic organisms in which they live and other species that use them as hosts. These relationships include fishes, sponges, seaweeds, cnidarians, polychaetes, bryozoans, crustaceans, mollusks, and other echinoderms. For this area, 22 associations were registered, including commensalism, epibionts and parasitism. This work constitutes the first report for the associations of *Eucidaris thouarsii* with Suberites aff. ficus, *E. thouarsii* with *Ophiothela mirabilis*, and Holothuria (Thymiosicia) impatiens with *Encheliophis vermicularis*. Associations of *Pentaceraster cumingi* with *Zenopontonia soror*, and *Ophionereis annulata* with Malmgreniella cf. variegata are new records for Colombia. This work also expands the range of hosts previously described for *Ophiothela mirabilis* and expands the distribution of the association between *Diadema mexicanum* and Echineulima cf. robusta.

## Introduction

Echinoderms are distributed in all coastal environments from tidal pools to rocky and coral reefs, in which they share space and refuge areas with members of their own phylum and other taxa (Sotelo-Casas and Rodríguez-Troncoso 2014). Due to this closeness, different types of interactions have been developed; within these associations are found the ones in which echinoderms depends on other organisms such as sponges and octocorals for protection against predators and for easy access to food (Henkel and Pawlik 2005, Marin et al. 2005). Others in which echinoderms interact with other organisms and the substrate, for example species that depend on the fixing cavities constructed by sea urchins such as *Echinometra lucunter lucunter* (Linnaeus, 1758) (Schoppe 1991).The cavities of this sea urchin, from the Caribbean Sea, are used by the brittle star *Ophiothrix synoecina* (Schoppe, 1996) which is obligated symbiont of *E. lucunter lucunter* (Schoppe 1996; Schoppe and Werding 1996). Finally, the mutualism occurring between different species of detritivorous sea cucumbers that share their inhabiting spaces and adopt different schedules for feeding and positions allowing other species to take advantage of the food (Rupert and Barnes 1996).

Relationships occur in all echinoderm classes; for example, the starfishes are frequently inhabited by symbionts of various taxonomic groups such as polychaetes, copepods, and mollusks (Jangoux 1990, Antokhina et al. 2012), with some species that are obligate symbionts (i.e., *Hololepidella millari*, *Doridicola echinasteris*) (Antokhina and Britayev 2012). For sea cucumbers, interactions with at least nine phyla have been described including diatoms, protozoans, flat worms (i.e., Xenacoelomorphos), polychaetes, mollusks, crustaceans, fish, and even other echinoderms (Jangoux 1990, Eeckhaut et al. 2004, Purcell et al. 2016). Related to sea urchins, different types of relationships have also been established, including commensalism with animals as the crab *Stenorhynchus debilis* (Smith, 1871) and a fish of the genus *Apogon* for which the sea urchin spines served as a refuge (Sotelo-Casas and Rodríguez-Troncoso 2014), and sponges that use the spines of sea urchins as an attachment substrate (Hétérier and De Ridder 2004, Aguirre et al. 2011). Although studies on crinoids are very limited, the association and dependence of many of the myxostomid species (Annelida) with this group has been recorded; approximately 100 of the 150 species of myxostomid currently described live above or within crinoids during their adult stage (Summers and Rouse 2014). For brittle stars, interactions with different kind of organisms have been reported, by having different adaptations in color and the brittle stars *Ophionereis* behavior to simulate the host, this is the case of the polynoid *Harmothroe lunulata*, *Ophionereis reticulata* and *O. annulata* (Millott 1953, Granja-Fernández et al. 2013, Gómez-Maduro and Díaz-Díaz 2017). Finally, many associations with benthic organisms such as sponges have been described in relation to ophiuroids (Bejarano et al. 2004, Marin et al. 2005), in some cases they depend specifically on other organisms for their development (Pardo et al. 1988).

The most studied marine groups related to their interactions with echinoderms are Mollusca and Crustacea. Mainly bivalve and gastropod relationships have been recognized (Caullery 1952), with more than 30 species of prosobranchs recorded as parasites of echinoderms (Caullery 1952), especially echinoids (Hyman 1955). It has also been found that most groups of crustaceans have some type of association with echinoderms, for example cirripedes are considered endo- and ectosymbionts of species such as *Dendrogaster* spp. (Caullery 1952), isopods have both obligate and non-specific relationships with all the five classes of echinoderms, and shrimps include species that are obligated commensals of some echinoderms (Ross 1983).

Relationships between echinoderms and different types of organisms have been widely registered throughout the world, but these are poorly studied and understood in Colombian waters and in the entire Tropical Eastern Pacific. To this end, relationships of the echinoderms with other phyla were recorded during a project developed to characterize the biodiversity of the rocky reefs of Chocó Norte in Colombia.

## Materials and methods

**Figure 1.**
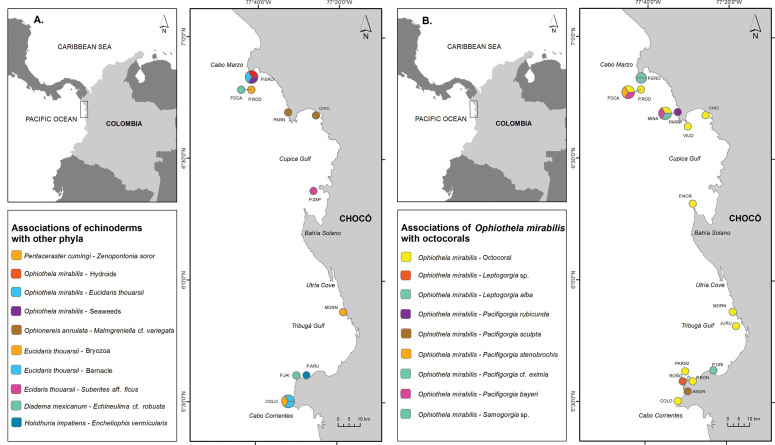
**A** Locations along northern Colombian Pacific (Chocó) where associations of echinoderms with other phyla were collected. Locations names from north to south P.ERO: Piedra de Eroito, FOCA: la foca, P.ROD: Piedra de Rodrigo, MINA: La mina, PARN: Parguera norte, VIUD: la Viuda, CHIC: Chicocora, P.ZAP: Piedra Zapata, P.NOR: Punta norte, MORN: Morromico norte, JURU: Jurubidá, P.ORI: Punta Orión, P.BON: Piedra bonita, PARS: Parguera sur, P.ARU: Punta Arusí, P.JAI: Piedra de Jairo, ROÑO: Roñosa, AMGR: Amargal, COLO: Coló. The line between FOCA and FARO represents the proximity between both stations. **B** Locations were associations between *O. mirabilis* and octocorals were registered. Locations names from north to south P.ERO: Piedra de Eroito, FOCA: la foca, P.ROD: Piedra de Rodrigo, MINA: La mina, PARN: Parguera norte, VIUD: la Viuda, CHIC: Chicocora, P.ZAP: Piedra Zapata, P.NOR: Punta norte, MORN: Morromico norte, JURU: Jurubidá, P.ORI: Punta Orión, P.BON: Piedra bonita, PARS: Parguera sur, P.ARU: Punta Arusí, P.JAI: Piedra de Jairo, ROÑO: Roñosa, AMGR: Amargal, COLO: Coló. The lines among several locations represent the proximity between them.

Individuals were collected during two expeditions carried out on April and October 2016 in the northern area of the Colombian Pacific, Chocó Department (Figure 1), between Cabo Corrientes in the south (5°29'N, 77°32'W) and Cabo Marzo in the north (6°49'N, 77°41'W). Those expeditions were performed to increase the knowledge in terms of diversity and distribution of the marine biodiversity of the rocky reefs of the area. In the Tropical Eastern Pacific, rocky reefs (called “Riscales” and “Morros” in Chocó), represent habitats for many invertebrate species, including corals, sea fans, and fishes, making them important and productive ecosystems for artisanal fishing (Rubio and Angulo 2003). Some of the rocky reefs are submerged, rising above the level of low tide or forming small permanently emerged islets (Díaz et al. 2016).

Samplings were made using scuba diving, and a direct and random collection of echinoderms was made by sweeping each station looking for all potential habitats in different areas of the rocky reef, at all depths starting at 25 m and finishing at 5 m, the top of the submerged reefs. All field information was recorded for each individual collected, including depth and habitat. The collected echinoderms were relaxed using magnesium chloride dissolved with sea water (MgCl_2_.6H_2_O) and fixed in 96% ethanol. Specimens were morphologically reviewed, photographed, and identified using stereoscope and microscopes. In order to correctly identify sea cucumbers, body wall ossicles were examined.

During the study, all the associated organisms were photographed in field, separated from the echinoderm when possible, and identified by expert taxonomists of each of the groups. The photographs taken in field were reviewed in order to complement the information of the associations, especially those that included octocorals to confirm the distribution range of the associations. Posterior samplings in the same area, carried out during 2017 and 2018 and focused mainly on octocoral biodiversity of the same locations, allowed us to expand the information on these relationships and are included in this work too.

All the collected material was deposited in the biological collections from the Museo de Historia Natural Marina de Colombia (**MHNMC**) – Makuriwa of INVEMAR.

## Results and discussion

A total of 22 relationships were registered between echinoderms and other organisms in the rocky reefs of the northern Chocó in the Colombian Pacific. These relationships include fish, sponges, cnidarians, polychaetes, bryozoans, crustaceans, and mollusks (Table 1). All the individuals were identified to the lowest possible taxonomic level, excepting some specimens that were registered only in photographs.

**Table 1.** Relationships between echinoderms and other marine groups found in the rocky reefs of northern Chocó, Colombian Pacific. Key for the relationships C: Commensalism, E: Epibiont, P: Parasitism. Key for Micro-habitats 1: Exposed in the Rocky reef, 2: Exposed in other living organisms, 3: Under rocks in contact with sand, 4: Partially exposed in the Rocky reef, 5: Partially exposed between rocks.

Echinoderm	Other organisms	Relationship	Micro-habitat	Depth (m)	Figure
***Pentaceraster cumingi***	*Zenopontonia soror*	C	1	19	Figure 2
***Ophiothela mirabilis***	*Eucidaris thouarsii*	E	2	7	Figure 3C
	*Leptogorgia alba*	C	2	3-19	Figure 3A
	*Leptogorgia* sp.	C	2	3-19	
	*Pacifigorgia adamsi*	C	2	7-19	Figure 3H
	*Pacifigorgia bayeri*	C	2	5-19	Figure 3C
	*Pacifigorgia eximia*	C	2	7-19	Figure 3E
	*Pacifigorgia irene*	C	2	7-19	Figure 3B
	*Pacifigorgia rubicunda*	C	2	15	Figure 3F
	*Pacifigorgia stenobrochis*	C	2	7-19	
	*Pacifigorgia sculpta*	C	2	25	
	*Samogorgia* sp.	C	2	7-19	
	*Muricea squarrosa*	C	2	7	
	Cnidarians	E	2	5	
	Seaweeds	E	2		
***Ophionereis annulata***	Malmgreniella cf. variegata	C	3	15	Figure 4
***Eucidaris thouarsii***	Bryozoans	E	4		Figure 5
	Suberites aff. ficus	E	5	9	Figure 5B
	Barnacles	E	4		Figure 5
	*Ophiothela mirabilis*	E	4		Figure 5C
***Diadema mexicanum***	Echineulima cf. robusta	P	4	19	Figure 6
***Holothuria impatiens***	*Encheliophis vermicularis*	P	3	7	Figure 7

### *Pentaceraster cumingi* (Gray, 1840) – *Zenopontonia soror* (Nobili, 1904)

Figure 2

**Figure 2.**
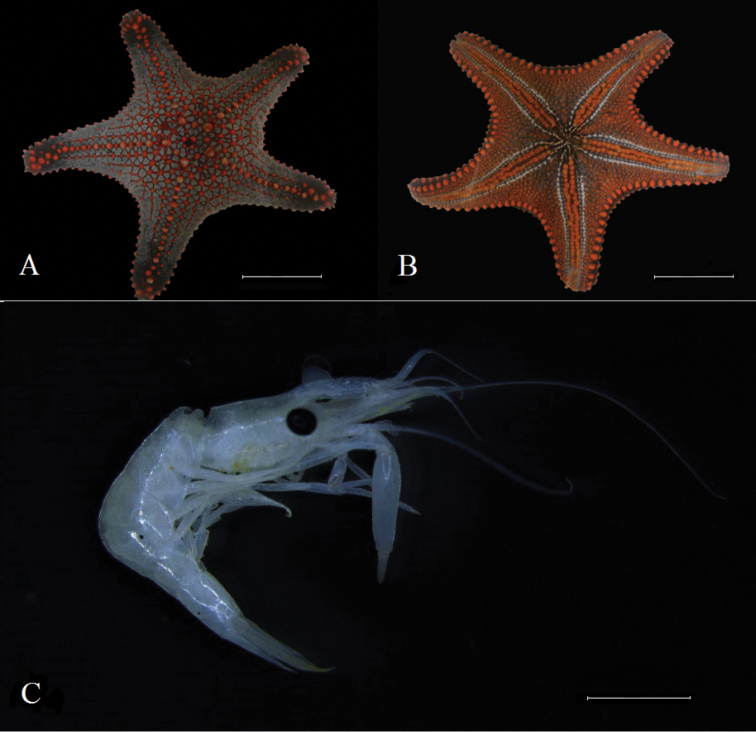
**A***Pentaceraster cumingi* aboral view **B***P. cumingi* oral view **C***Zenopontonia soror*. The shrimp lost the color after fixing in 96% ethanol. Scale bars: 100 mm (**A, B**), 2 mm (**C**).

**Material**: one specimen of *Pentaceraster cumingi* (INV EQU4283) was collected with two shrimps (*Zenopontonia soror*) located in the oral part (Figure 2). Shrimps were orange, the same color that the ambulacral feet of the sea star. This relationship was recorded in Piedra de Rodrigo (P.ROD) (Figure 1).

Starfish are frequently inhabited by several symbiotic animals (Jangoux 1990). Within the genus *Pentaceraster* the association with *Zenopontonia soror* has been commonly reported world-wide. This commensal shrimp belongs to the family Palaemonidae and is known for being a specialized and obligate symbiont of starfishes (Antokhina and Britayev 2012). *Zenopontonia soror* was initially described in the Red Sea (Nobili 1904), but Edmonson (1935) and Gordon (1939) reported its association with some species of asteroids of the Indo-Pacific, and finally Bruce (1976) reported it in Australia associated with *Plectaster decanus* (Müller & Troschel, 1843) and in Pacific of Panama with *Pentaceraster cumingi* (as *Oreaster occidentalis*) (Bruce 1976), both sea stars being common in reefs. *Zenopontonia soror* is currently reported associated with 21 asteroids species, including five species of the genus *Pentaceraster* (Antokhina and Britayev 2012). This is the first report of the association between *Zenopontonia soror* and *Pentaceraster cumingi* for the Colombian Pacific.

### *Ophiothela mirabilis* Verrill, 1867 and several symbiosis associations

Figure 3

**Figure 3.**
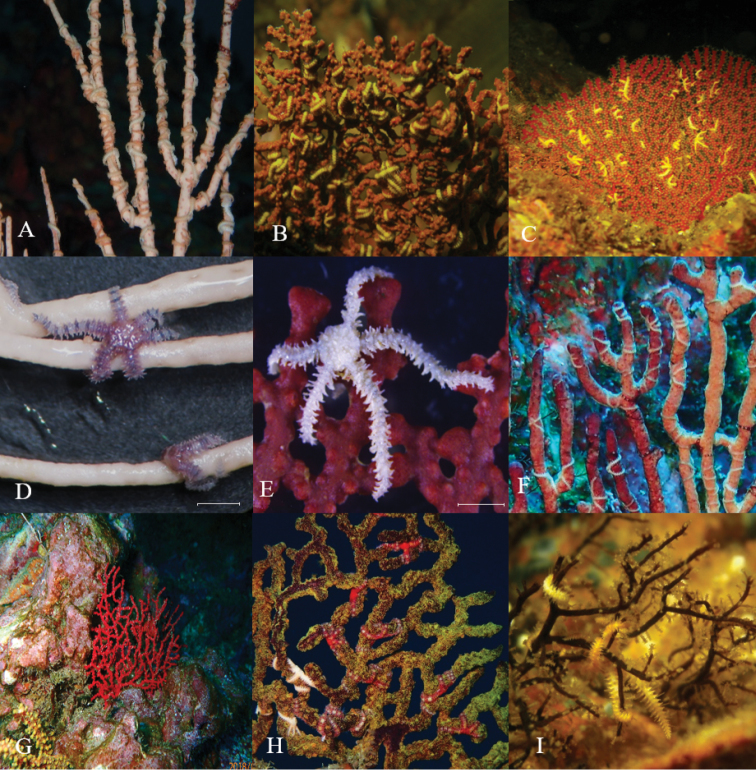
*Ophiothela mirabilis* on different cnidarians **A***Leptogorgia alba***B***Pacifigorgia rubicunda***C***P. eximia***D***Ophiothela mirabilis* collected on *L. alba***E***O. mirabilis* collected on *Pacifigorgia irene***F***P. stenobrochis***G***P. sculpta***H***P. bayeri***I** Cnidarian. Scale bars: 2 mm (**D, E**).

**Material**: *Ophiothela mirabilis* was registered on different hosts: *Eucidaris thouarsii* (INV EQU4218) (Figure 5C), cnidarians (Figure 3I), seaweeds, and octocorals: *Leptogorgia alba* (Duchassaing y Michelotti, 1864) (INV EQU4251) (Figure 3A, D), *Leptogorgia* sp., *Pacifigorgia rubicunda* Breedy y Guzman, 2003 (Figure 3B), *Pacifigorgia eximia* (Verrill, 1868) (Figure 3C), *Pacifigorgia irene* Bayer, 1951 (Figure 3E), *Pacifigorgia stenobrochis* (Valenciennes, 1846) (Figure 3F), *Pacifigorgia sculpta* Breedy & Guzman, 2004 (Figure 3G), *Pacifigorgia bayeri* Breedy, 2001 (Figure 3H), *Pacifigorgia adamsi* (Verrill, 1868), *Samogorgia* sp. and *Muricea squarrosa* Verrill, 1869. One cnidarian morphotype, possibly a hydrozoan, and the seaweeds could not be identified because they were not collected. *Ophiothela mirabilis*’ relationship with octocorals was observed in 16 stations during April (2016) including Chicocora (CHIC), la Foca (FOCA), la Mina (MINA), Parguera (PARN), Piedra de Eroito (P.ERO), Punta norte (P.NOR), Piedra de Rodrigo (P.ROD), la viuda (VIUD), Coló (COLO), Morromico norte (MORN), Jurubidá (JURU), la Roñosa (ROÑO), Amargal (AMGR), Parguera norte (PARN2), Piedra bonita (P.BON) and Punta Orión (P.ORI). In October (2016) no octocoral was registered with this brittle star; however, the relationship was observed in subsequent samplings developed in October 2017 (not included on this work) and 2018.

Brittle stars are usually associated with organisms such as sponges, cnidarians, sea urchins, and even algae which provide shelter and a place to feed (Bejarano et al. 2004, Hendler et al. 2012). Some authors like Neira and Cantera (2005) and Lawley et al. (2018) (as Ophiothela cf. mirabilis) have found that *O. mirabilis* has a preference for octocoral hosts. However, Mantelatto et al. (2016) indicated that in Brazil, area where it is invasive, *O. mirabilis* is an opportunistic and generalist species in relation to host species selection, finding it related to 20 different invertebrates (i.e., *Dysidea etheria* – sponge, *Isostichopus badionotus* – sea cucumber). The relationship between this brittle star and its hosts is considered as commensalism; however, it has been suggested that there may be a negative effect to the host due to the high densities that *O. mirabilis* can present (Mantelatto et al. 2016). Those negative effects may not be directly caused by its feeding on the host, but some authors suggest that it may be related to structural damage due to the increase in the weight of commensals that the host must support, and in the case of the cnidarians with the possible interruption of the extension of the polyps making it difficult to feed and compromising the ability of the octocoral to obtain nutrients (Mantelatto et al. 2016, Thé de Araújo et al. 2018). In northern Chocó, high densities of *O. mirabilis* on the octocorals were observed during April 2016 and in subsequent samplings in 2017 and 2018, but further studies are needed to elucidate the possible negative effect of this relationship to the octocorals in this area. For the Colombian Pacific, *O. mirabilis* has been reported to be associated mainly with the octocoral *Leptogorgia alba* (Cantera et al. 1987, Pardo et al. 1988, Neira and Cantera 2005). However, the results presented here expand its range of hosts to include other octocoral species: *Pacifigorgia rubicunda*, *P. adamsi*, *P. eximia*, *P. irene*, *P. bayeri*, *P. sculpta*, *P. stenobrochis*, *Muricea squarrosa*, *Samogorgia* sp., and *Leptogorgia* sp., leafy algae, and *E. thouarsii*.

Several individuals of *O. mirabilis* were found using *E. thouarsii* spines as a fixing substrate, but there are no studies of *O. mirabilis* as a sea urchin epibiont in the Tropical Eastern Pacific; however, for the Brazilian Caribbean, where is an invasive species, *O. mirabilis*, has been reported living in high densities in *Echinometra lucunter* spines (Mantelatto et al. 2016). This constitutes the first report of the relationship between *O. mirabilis* and *E. thouarsii*, expanding the range of hosts for *O. mirabilis*.

Beside the groups mentioned above, others organisms have also been reported as *O. mirabilis* hosts, especially in the Mexican Pacific where association with scleractinian corals and sponges have been reported (Granja-Fernández and López-Pérez 2011), and in the Caribbean (Brazil), others groups were found including sponges, ascidians, and bryozoans (Mantelatto et al. 2016). So far there are no other reports of *O. mirabilis* living in seaweeds. Although the species of this group was not identified to species level, this would be the first record of the relationship between *O. mirabilis* and seaweeds.

### *Ophionereis annulata* (Le Conte, 1851) – Malmgreniella cf. variegata

Figure 4

**Figure 4.**
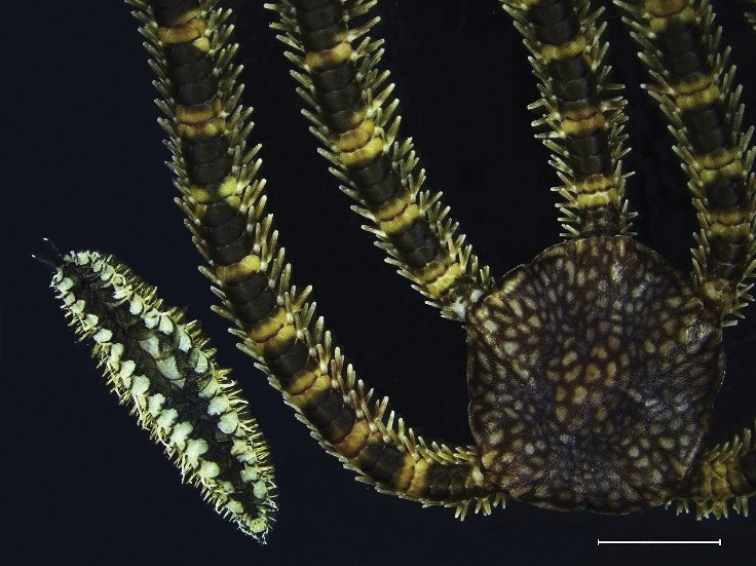
Malmgreniella cf. variegata (left) and *Ophionereis annulata* (right). Scale bar: 5 mm.

**Material**: two polychaetes of the family Polynoidae identified as Malmgreniella cf. variegata were found living on specimens of *Ophionereis annulata* collected in Chicocora (CHIC) (INV EQU4370) and Parguera (PARN) (INV EQU4208) (Figure 1). The polychaetes had a similar coloration pattern to the dorsal side of the ophiuroid arms (Figure 4).

The association between polychaetes of the family Polynoidae with brittle stars belonging to the genus *Ophionereis* has been reported for the Pacific Ocean and the Caribbean Sea. *Malmgreniella variegata* (Treadwell, 1917) distributed mainly in the western Atlantic, including the Caribbean Sea (GBIF 2019, OBIS 2018) has a relationship with *Ophionereis reticulata* (Say, 1825) (Hendler et al. 1995). This association has been well described for Brazil (Santa-Isabel et al. 1996) and recently for Venezuela (Gómez-Maduro and Díaz-Díaz 2017). *Malmgreniella variegata* has a similar coloration and a banded pattern as the ophiuroid arms, which allows it to go unnoticed on the host (Pettibone 1993, Gómez-Maduro and Díaz-Díaz 2017). Malmgreniella cf. variegata has been reported mainly associated with the aboral part of the disk of the ophiuroid, using it as a refuge (Granja-Fernández et al. 2013). Although *M. variegata* is distributed in the Atlantic Ocean, some authors such as Pettibone (1993) reported the species living on *Ophionereis annulata* in the Gulf of Panama. In the Mexican Pacific, Granja-Fernández et al. (2013) described a similar commensal relationship between *O. annulata* and a polynoid polychaete, which, after reviewing its morphology, was identified as M. cf. variegata, due to differences in the color pattern of the elytra and the notochaetae from *M. variegata* from the Caribbean Sea. After examination of the specimens collected in northern Chocó we found all the taxonomic characteristics described by Salazar-Silva (2009) for the Atlantic polychaete *Malmgreniella variegata* except for the color pattern of the elytra, similar to the results described by Granja-Fernández et al. (2013). The identification of these polychaetes needs to be revised using additional evidence, such as molecular analysis. This is the first report of the presence of M. cf. variegata and its association with *O. annulata* in the Colombian Pacific.

### *Eucidaris thouarsii* (L. Agassiz y Desor, 1846) – Bryozoa, Cirripedia, Suberites aff. ficus, and *Ophiothela mirabilis* Verrill, 1867

Figure 5

**Figure 5.**
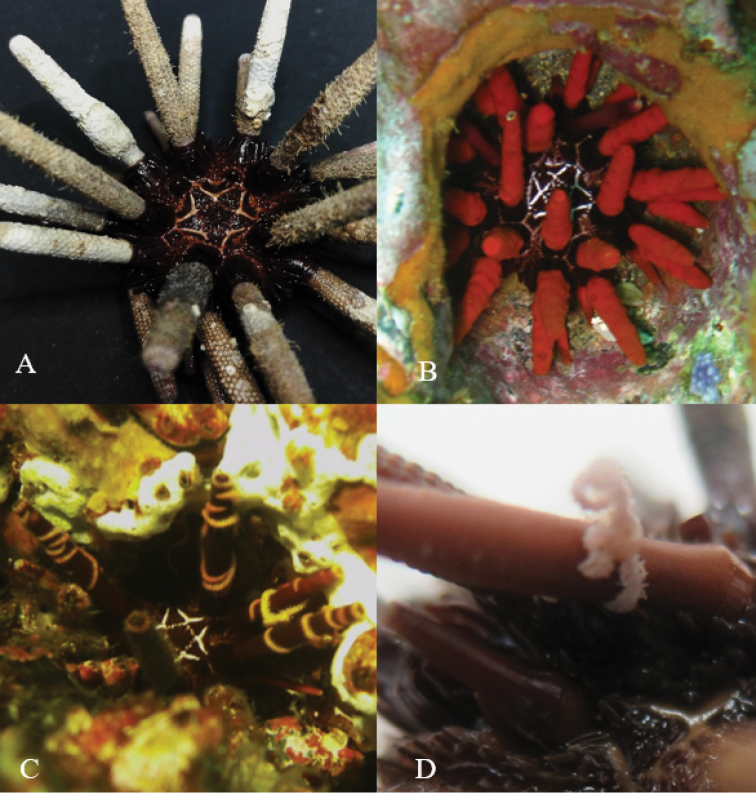
Epibionts on *Eucidaris thouarsii* spines **A** Bryozoans **B**Suberites aff. ficus**C***Ophiothela mirabilis***D** close up to *O. mirabilis* collected from *E. thouarsii* spine.

**Material**: Four types of epibionts were found inhabiting on *Eucidaris thouarsii* spines in different stations (Figure 1), including cirripede barnacles (INV EQU4218), several species of bryozoans (INV EQU4528, INV EQU4293, INV EQU4299) (Figure 5A), and the sponge Suberites aff. ficus (INV EQU4301) in Piedra Zapata (P.ZAP) (Figure 5B). The ophiuroid *O. mirabilis* was collected on the sea urchin (INV EQU4218) in Coló (COLO) and Piedra de Eroito (P.ERO) (Figure 5C, D).

Only one sea urchin morphotype belonging to the family Cidaridae was found in the rocky reefs in northern Chocó, identified as *Eucidaris thouarsii*, mostly due to its thick spines (Figure 5). Different organisms use *E. thouarsii* spines as an attachment substrate, including sessile (bryozoans, sponges) and mobile animals (*O. mirabilis*). There are not many published papers related to the epibionts species of sea urchins; however, it has been reported that sponges, especially species belonging to the genus *Clathria*, grow on the spines and the discs of *E. thouarsii* (David et al. 2009, Aguirre et al. 2011). For other species of sea urchin, different associations have been reported: for *H. asteriscus* more than 20 species of epifauna have been identified, including nine species of polychaetes, five species of bryozoans, three species of mollusks, three species of crustaceans, two sponge species, and a single species each of protozoan, cnidarian, nematode, and echinoderm (Salazar and López 1983). The reason sea urchins of the family Cidaridae are commonly used as hosts is that they have spines made of muscle and collagen in their basal part which allows the settlement of epibiont fauna, unlike other echinoids which have antifouling compounds (David et al. 2009, Aguirre et al. 2011). In this work, the sponge that settled on *E. thouarsii* spines was identified as Suberites aff. ficus (Figure 5B) because of the spicules. *Suberites ficus* (Johnston, 1842) is the given name for a complex of species with megascleres, tylostyles, microscleres, strongyles, and oxeas, originally from the North Atlantic Ocean but with two records on the eastern Pacific (Dickinson 1945, Bakus and Green 1987). The tylostyles and microscleres from the Colombian Pacific specimen are smaller (tylostyles: 90–221 × 3–8 µm; microscleres: 17–42 × 1–3 µm) than the ones reported in specimens from other areas of the Eastern Pacific, such as lower California, Mexico (tylostyles: 340 × 10 µm; microscleres: 18–36 × 1–3 µm; De Laubenfels 1932) and south California (tylostyles: 120–680 × 1–11 µm; microscleres: 20–48 × 1–2.5 µm; Bakus and Green 1987). Until now, *Clathria* was the only sponge genus reported growing on *E. thouarsii* (Aguirre et al. 2011), but *S. ficus* has been reported living on other organisms such as gastropod shells (Bakus and Green 1987). This report constitutes the first record of S. aff. ficus and its association with *E. thouarsii* for the Colombian Pacific. At least five unidentified bryozoan species (Figure 5A) have been observed on the spines of *E. thouarsii*, which were collected in parts of the study area (INV EQU4528, INV EQU4293, INV EQU4299).

The phylum Crustacea is another of the groups reportedly associated with sea urchins; different species, especially of crabs and shrimps, have been found living between the spines and, in some cases, attached to the spines (Macía and Robinson 2012, Britayev et al. 2013), using the spines as protection and on occasion benefiting from the sea urchin’s grazing (Morton 1988). However, there is not much information about other crustaceans, especially about barnacles symbiotic with sea urchins. Some species that have been reported living in sea urchin spines are *Balanus trigonus* and *Paralepas percarinata* (as *Heteralepas percarinata*) (Werner 1967, Aldous 1970).

### *Diadema mexicanum* A. Agassiz, 1863 – Echineulima cf. robusta (Pease, 1860)

Figure 6

**Figure 6.**
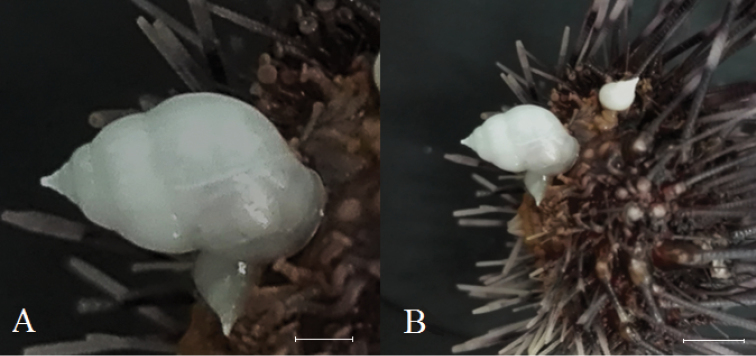
**A**Echineulima cf. robusta**B***Diadema mexicanum* (INV EQU4292) with three specimens of Echineulima cf. robusta. Scale bars: 2 mm (**A**), 4 mm (**B**).

**Material**: Three specimens of Echineulima cf. robusta were found on the oral portion of one specimen of the sea urchin *Diadema mexicanum* (INV EQU4292), and five organisms were found on another specimen (INV EQU4530). Both sea urchins were juveniles (INV EQU4292, 18.6 mm test diameter; INV EQU4530, 25.4 mm test diameter). The specimens were collected in Piedra de Jairo (P.JAI) (INV EQU4292) and La Foca (FOCA) (INV EQU4530) (Figure 1). The gastropods were white in color (Figure 6).

The associations between echinoderms and gastropods have been widely recorded for the family Eulimidae, including 750 species (Warén 1983, Jangoux 1984). For the species *Centrosthepanus coronatus* it was reported that they have an ectoparasitic relationship with gastropods of the genus *Echineulima* (Jangoux 1984, Cantera and Neira 1987). These gastropods parasitize the interambulacral zone on the oral side of the test, using a proboscis that penetrates the skeleton to feed on the hemal fluid of its host (Cantera and Neira 1987). This relationship is considered as parasitism because of the damage caused by the gastropod to the sea urchin; among the negative effects that have been recorded for these gastropods, in addition to the fact that they feed on the sea urchin, they produce lesions by their grip, causing dermal swellings and even distortion in the skeleton (Jangoux 1984). In the present work two individuals of *Diadema mexicanum* were collected with several individuals of Echineulima cf. robusta present on the oral side, close to the mouth (Figure 6). *Echineulima robusta* has already been registered as parasite of the sea urchins of the same family in the Indo-Pacific (Warén 1983); however, for the Colombian Pacific Cantera and Neira (1987) reported *Echineulima* sp. parasitizing *C. coronatus* for Gorgona Island. Other species of *Echineulima* have been reported parasitizing sea urchins in other geographical areas, such as in the Gulf of California (Mexico) and Taboga islands (Panama) where the association between *D. mexicanum* and *Echineulima mittrei* (Petit de la Saussaye, 1851) has been observed (Lützen and Nielsen 1975, Alvarado et al. 2015), and in Hawaii where *Echineulima thanuumi* (Pilsbry, 1921) has been reported parasitizing sea urchins of the genus *Echinotrix* (Cantera and Neira 1987). Olivares-González (1986) found that there exists a preference of the parasite *E. mittrei* for sea urchins with test diameters of approximately 20 mm to 39 mm, although the gastropod was found in sizes ranging from 20 mm to 60 mm. These preferences are related to the energy expenditure; in other words, sea urchins smaller than 20 mm use their energy for body growth while larger animals use it to produce gametic material, which is the presumably the preferred source of food for the gastropod.

Three specimens of Echineulima cf. robusta were found on one of the sea urchins of *Diadema mexicanum*, and five on the other; however, in this last one the gastropods were found in the oral side of the test separated in what looked like two different groups: the first group had three individuals, one of them bigger than the other two, and the other group had two individuals similar in size. Olivares-González (1986) found that sea urchins with more than one individual have them organized in groups of one female and one or two males; the males sharing a single impression on the test but with different apertures. Although the association between gastropods of the genus *Echineulima* with other sea urchins has already been reported for Gorgona Island (Cantera and Neira 1987), this report constitutes the first report for the Colombian Pacific of Echineulima cf. robusta and its relationship with *Diadema mexicanum*. Additionally, in this work we are reporting that Echineulima cf. robusta can parasitize smaller sea urchins (18.6 mm test diameter) than previously reported.

### Holothuria (Thymiosycia) impatiens (Forskål, 1775) – *Encheliophis vermicularis* Müller, 1842

Figure 7

**Figure 7.**
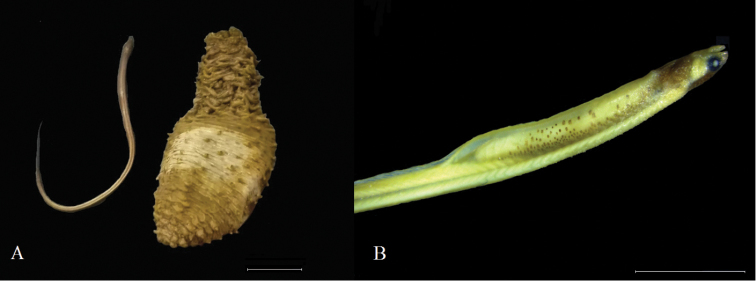
**A***Holothuria impatiens* (right) and *Encheliophis vermicularis* (left) **B** Close up of *E. vermicularis*. Scale bars: 8 mm (**A**), 5 mm (**B**).

**Material**: One specimen of *Encheliophis vermicularis* (Figure 7) was found inside of one specimen of Holothuria (Thymiosycia) impatiens (INV EQU4240), collected in Punta Arusí (P.ARU) (Figure 1). The fish measured 58.76 mm length.

The family Holothuriidae serves as host to several species of pearl fish of the family Carapidae (Luciano et al. 2002). In this family it has been found that the genera *Onuxodon* and *Carapus* act as commensals of the sea cucumbers but feed outside their hosts (Parmentier and Vandewalle 2003, Parmentier et al. 2016), while the genus *Encheliophis* behaves like a parasite, staying and feeding on internal tissues, causing small internal wounds and reducing the gonadal tissues of the host (Parmentier and Das 2004, Parmentier et al. 2006). In this work a specimen of *Encheliophis vermicularis* was found inside of Holothuria (Thymiosicia) impatiens. These fish usually dwell in the respiratory or digestive cavities of sea cucumbers, using them as protection and as a source of food (Trott 1981, Luciano et al. 2002, González-Wangüemert et al. 2014), and in some cases where pearl fish are found in pairs, the sea cucumber also serves as a breeding site (González-Wangüemert et al. 2014). The relationship between the genus *Encheliophis* with sea cucumbers has been reported for species such as Holothuria (Microthele) fuscopunctata Jaeger, 1833, Holothuria (Holothuria) tubulosa Gmelin, 1791, and *Isostichopus fuscus* (Ludwig, 1875), amongst others (Parmentier and Vandewalle 2005, Purcell et al. 2016). Regarding *E. vermicularis*, some authors found that it has a very specific relationship with Holothuria (Mertensiothuria) leucospilota (Brandt, 1835) (Miyazaki et al. 2014), Holothuria (Mertensiothuria) hilla Lesson, 1830 (James 1995) and with lower incidence with Holothuria (Halodeima) atra Jaeger, 1833 (Smith 1964). Although this fish species has already been reported for Gorgona Island as *Encheliophis hancocki* (Reid, 1940) (Reid 1940, Orrell and Hollowell 2018), a synonymized name of *E. vermicularis* (Froese and Pauly 2018), this work expands its distribution to the northern Chocó.

## Conclusions

Despite echinoderms constituting an important group with representatives present in all marine ecosystems, and many different kinds of interaction with other phyla have been described, the information on their associations for the Colombian Pacific was limited. This work has helped to increase the knowledge on echinoderms and their associations with other groups including fishes, mollusks, polychaetes, cnidarians, and sponges from the rocky reefs of the Colombian Pacific and in general from the Tropical Eastern Pacific.

## Ethics approval and consent to participate

These species do not appear as evaluated in the IUCN Red List. Riscales project is part of the Biodiversity and Marine Ecosystems research program of the Instituto de Investigaciones Marinas y Costeras of Colombia (INVEMAR), which belongs to the Ministry of Environment and Sustainable Development of Colombia, in accordance with Law 99 of 1993 (Article 18), which does not require Permission of Collection of specimens for Scientific Research according to the decree 1076 of 2015 (Chapter 8, Section 1, Article 2.2.2.8.1.2., Paragraph 1).
